# Improved Registration Algorithm Based on Double Threshold Feature Extraction and Distance Disparity Matrix

**DOI:** 10.3390/s22176525

**Published:** 2022-08-30

**Authors:** Biao Wang, Jie Zhou, Yan Huang, Yonghong Wang, Bin Huang

**Affiliations:** School of Instrument Science and Opto-Electronics Engineering, Hefei University of Technology, Hefei 230009, China

**Keywords:** point cloud registration, machine vision, feature extraction, double threshold, distance disparity matrix, ICP algorithm

## Abstract

Entire surface point clouds in complex objects cannot be captured in a single direction by using noncontact measurement methods, such as machine vision; therefore, different direction point clouds should be obtained and registered. However, high efficiency and precision are crucial for registration methods when dealing with huge number of point clouds. To solve this problem, an improved registration algorithm based on double threshold feature extraction and distance disparity matrix (DDM) is proposed in this study. Firstly, feature points are extracted with double thresholds using normal vectors and curvature to reduce the number of points. Secondly, a fast point feature histogram is established to describe the feature points and obtain the initial corresponding point pairs. Thirdly, obviously wrong corresponding point pairs are eliminated as much as possible by analysing the Euclidean invariant features of rigid body transformation combined with the DDM algorithm. Finally, the sample consensus initial alignment and the iterative closest point algorithms are used to complete the registration. Experimental results show that the proposed algorithm can quickly process large data point clouds and achieve efficient and precise matching of target objects. It can be used to improve the efficiency and precision of registration in distributed or mobile 3D measurement systems.

## 1. Introduction

With the rapid development of machine vision in recent years, vision technology based on 3D point clouds has been widely used in the fields of industrial design, reverse engineering, surface defect detection, and virtual reality. Compared with traditional 2D images, 3D data provide richer information [1]. As a special information format that contains complete 3D spatial data, 3D point cloud data have elicited extensive attention [2]. At present, methods for collecting 3D data include the time-of-flight [3], stereo vision [4], laser scanning [5], and structured light [6] methods. Limited by the scanning angle of the device and the shape of the object, the complete 3D information of an object must be collected from multiple views, and point clouds must be registered into a complete model. Point cloud registration is a key step in capturing the complete shape of 3D objects.

The purpose of point cloud registration is to find a 3D rigid body transformation, such that the point cloud of the same object from different perspectives can be transformed into the same coordinate system for rapid and accurate matching and splicing. Splicing accuracy directly affects the accuracy of model reconstruction [7]. The same-source registration can be divided into optimization-based registration methods, feature-learning methods, and end-to-end learning registration [8]. The deep learning-based methods do not require iteration, but large training data is needed [9,10,11]. Besides, the registration results are sensitive to noise. Optimization-based registration is to use optimization strategies to estimate the transformation matrix without training data. The most widely used point cloud registration algorithm is the iterative closest point (ICP) algorithm proposed by Besl et al. [12]. This algorithm requires a good initial position and a high overlap rate. Moreover, it easily falls into the local optimal solution. Therefore, this algorithm is typically used for fine registration [13]. To improve the efficiency and accuracy of registration, scholars have proposed a variety of methods, including velocity updating ICP [14], generalised ICP (GICP) [15], and globally optimal ICP [16]. Magnusson [17] proposed a registration algorithm that differed from the ICP registration model, called the 3D normal distribution transformation algorithm. This algorithm was based on the probability density model, did not require calculating the nearest neighbour corresponding points, and reduced computational complexity. Zhong [18] designed an inherent shape feature to describe point feature information by establishing the local reference coordinate system and 3D histogram of each point. However, the uneven distribution of points and measurement noise affected the registration results. Rusu et al. [19] proposed a point feature histogram (PFH) algorithm and a fast PFH (FPFH) algorithm [20,21] for local feature descriptions. Yun et al. [22] calculated congruent triangles by using the centroid of a point cloud ball to obtain the corresponding relationship between them. Coarse registration was performed through the centroid. Jauer P. et al. [23] used knowledge in mechanics and thermodynamics to assume that a point cloud is a rigid body composed of particles. A force was applied between two particle systems to cause one of the systems to move towards the rigid body of the other system to complete point cloud registration. Chen et al. [24] designed a novel descriptor based on a plane/line. This descriptor was particularly used for establishing structure-level correspondence between point clouds for coarse registration. Raobo Li et al. [25] proposed a point cloud registration method that used the feature transformation processing of the centre of gravity. Two feature vectors were constructed from the nearest and farthest points of the centre of gravity, and a third feature vector was synthesised. Finally, point cloud registration was performed. Wang et al. [26] used the principal component analysis (PCA) method to realise the coarse registration of point clouds. In fine registration, the nearest point is iterated on the basis of the two-way distance proportion. This method exhibits a certain improvement in accuracy and speed compared with the classical ICP algorithm; however, it is more sensitive to noise points. Song et al. [27] conducted a down-sampling of a point cloud, extracted feature points through the angle characteristics of a normal vector neighbourhood, and established FPFH for feature descriptions. The combination of the sample consensus initial alignment (SAC-IA) and ICP algorithms was used to complete point cloud registration. Xu et al. [28] proposed an improved ICP algorithm that combined the random sample consensus algorithm, intrinsic shape signatures, and 3D shape context. Min et al. [29] synthesized a hybrid mixture probabilistic model with the directional and positional information of each point to completes point cloud registration. KOIDE et al. [30] proposed a multi-point distribution aggregation method to extend the GICP approach.

The aforementioned methods can be classified into two categories. The first category includes algorithms based on a global search strategy, which reaches the optimal solution with continuous iteration. Their limitation is the existence of noise points, which increase computational costs. The second category includes methods based on feature matching, which extracts representative feature points from a point cloud to reduce the amount of calculation. The selection of feature points and their description method will determine the registration result. These methods frequently lead to low registration accuracy due to the lack of representativeness or insufficient number of feature points. The accuracy of feature point extraction directly affects registration precision. Therefore, studying an appropriate method to obtain accurate feature points is a challenge.

In the current study, we use the normal vector and the curvature of a local point cloud for feature extraction with double threshold to extract representative feature points. The FPFH descriptor is used to describe the feature points and obtain the initial corresponding point set. By considering the invariance of distance and angle between feature points, the distance disparity matrix (DDM) algorithm is used to eliminate the wrong corresponding point pairs, and thus, determine accurate corresponding point pairs. Then, the SAC-IA algorithm is used for coarse registration to obtain a better initial position of point clouds. Finally, the ICP algorithm is used for fine registration. Representative feature points are obtained by extracting feature points with double thresholds and removing mismatched pairs, making the subsequent registration fast and accurate. The combination of coarse and fine registration solves the problems of slowness and low accuracy of the ICP algorithm.

The remainder of this paper is organised as follows. Section 2 provides details of our proposed method and describes relevant principles. Section 3 introduces an experiment of this method on three models in the basic geometry library and evaluates its accuracy and efficiency. Section 4 presents the conclusions of this study.

## 2. Materials and Methods

**Figure 1:**
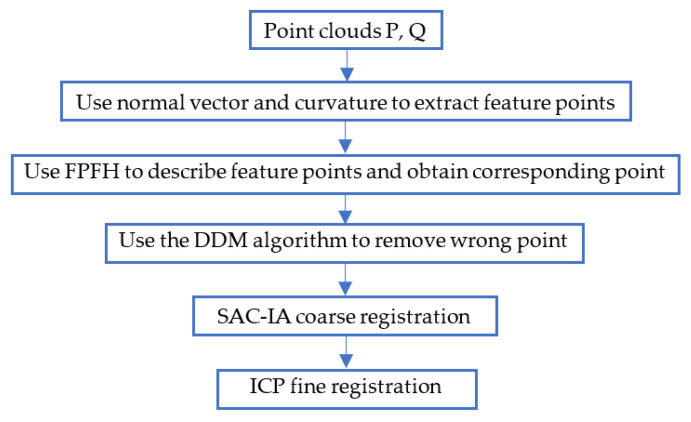
Flowchart of the proposed algorithm.

The flowchart of the proposed method, which mainly includes five steps, is shown in Figure 1. P represents the source point cloud, and Q represents the target point cloud. Firstly, the feature points of the origin cloud are extracted using the normal vector and curvature with double threshold to reduce the number of point clouds and registration time. Secondly, the FPFH is used to describe the feature points to obtain the initial set of corresponding point pairs. Thirdly, the mismatched point pairs are removed using the DDM algorithm to improve the accuracy of the corresponding point pairs. Fourthly, the SAC-IA algorithm is used for the coarse registration of point clouds to provide a good initial position for subsequent registration. Finally, the ICP algorithm is used for fine registration.

### 2.1. Normal Vector Calculation

**Figure 2:**
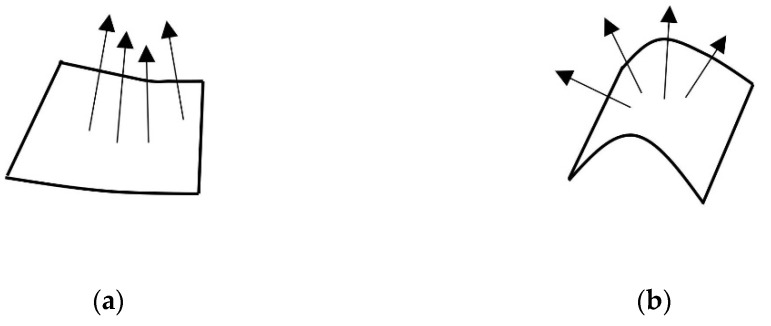
Normal vector reaction surface information. (**a**) Gradual region; (**b**) Abrupt region.

The normal vector is an important geometric attribute in a point cloud data model. The included angle of the normal vector of the points in the local neighbourhood can reflect the change information of the surface, as illustrated in Figure 2. When the normal vector in the region surface changes gradually, the region is relatively flat. When the normal vector changes abruptly, the region fluctuates considerably. Therefore, the appropriate threshold can be set in accordance with the change in the normal vector in the neighbourhood to obtain the feature point.

PCA [31] is a common method for calculating the normal vector. For any $p_{i}$ in point cloud P, covariance analysis is performed on $p_{i}$ and its neighbouring points $p_{ij}$ in its *k*-neighbourhood. The specific steps for calculating the normal vector are as follows:

(1) For point $p_{i}$($x_{i},y_{i},z_{i}$), its neighbourhood points $p_{i1}$, $p_{i2}$, … $p_{ik}$ are found in a ball of radius r.
(2) Equations (1) and (2) are used to calculate the normal vector of point $n_{i}$, where ${\bar{p}}_{i}$ is the 3D centroid of the neighbourhood point set, and C is the covariance matrix of ${\bar{p}}_{i}$. The three eigenvectors and the corresponding three eigenvalues of covariance matrix C are calculated via eigenvalue decomposition. The eigenvector that corresponds to the smallest eigenvalue is the normal vector of point ${\bar{p}}_{i}$.
(3) The direction of the normal vector is determined using Equation (3). In general, the direction of the normal vector is consistent towards the viewpoint direction (from viewpoint$\;v_{p}$ to $p_{i}$).

(1) $${\bar{p}}_{i}=\frac{1}{k}\sum_{j=1}^{k}p_{ij}$$

(2) $$C=\frac{1}{k}\sum_{j=1}^{k}\left(p_{ij}-{\bar{p}}_{i}\right){\left(p_{ij}-{\bar{p}}_{i}\right)}^{T}$$

(3) $$n_{i}=\{\begin{array}{c} n_{i},\;\;n_{i}\cdot \left(v_{p}-p_{i}\right)>0\\ -n_{i},\;\;n_{i}\cdot \left(v_{p}-p_{i}\right)<0 \end{array}$$

### 2.2. Curvature Calculation

**Figure 3:**
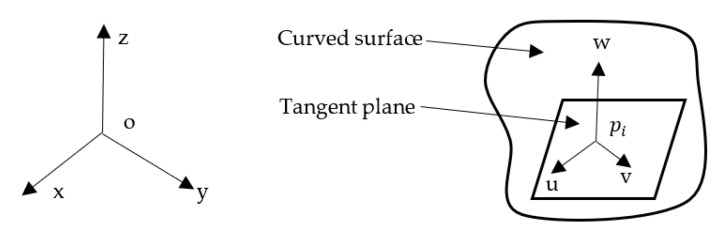
Local coordinate system.

Curvature is a concept that describes the degree of curvature of a surface and is a basic attribute of a surface. During point cloud processing, the curvature is also an important attribute for describing the geometric characteristics of point clouds. Each point in the point cloud and its adjacent points can be typically fitted into a local surface, and the local surface curvature is used as the curvature of the point. In the current study, the least squares method is used to calculate the curvature of point clouds. The curvature calculation process is summarised as follows:

(1) Point $p_{i}$ is set as the coordinate origin, establishing the local coordinate system (u, v, w). The direction of the normal vector of the surface at point $p_{i}$ is the direction of the w-axis. The u- and v- axes are on the tangent plane at point $p_{i}$, as shown in Figure 3. The u- v- and w-axes are orthogonal.
(2) Basic equation of quadric surface [32]:

(4) S(u,v)=(u,v,w(u,v))

(5) $$w\left(u,v\right)=au^{2}+buv+cv^{2}+eu+fv$$

If e and f in Equation (5) are equal to zero, then Equation (4) represents a quadratic parabolic surface. For point $p_{i}$, its neighbourhood point $p_{j}$ (j=1,2,…,k) is converted into the local coordinate system (u, v, w), and the converted coordinates are $\left(u_{j},v_{j},w_{j}\right)$. The coordinates are substituted into the formula calculation. When k is greater than 3, a set of overdetermined equations is obtained. In accordance with the least squares method, the optimal fitting parameters a, b, and c are finally obtained.

(3) The first fundamental quantity (E,F,G) and the second fundamental quantity (L,M,N) of the fitted surface can be obtained by solving the first- and second-order partial derivatives of the equation from the basic equation of the surface. Combined with the surface parameter equation, the principal curvature ($k_{1}$, $k_{2}$), Gaussian curvature (K), and average curvature (H) can be calculated.

(6) $$\{\begin{array}{c} k_{1}=a+c+\sqrt{{\left(a-c\right)}^{2}+b^{2}}\\ k_{2}=a+c-\sqrt{{\left(a-c\right)}^{2}+b^{2}}\\ K=k_{1}.k_{2}=\frac{LN-M^{2}}{EG-F^{2}}=4ac-b^{2}\\ H=\frac{k_{1}+k_{2}}{2}=\frac{EN-2FM+LG}{2\left(EG-F^{2}\right)}=a+c \end{array}$$

The feature extraction method based on curvature can accurately identify the feature information of a point cloud, accurately extract the detailed features of the abrupt and gradual regions of the surface and effectively retain the feature information of the model.

### 2.3. Double Threshold Feature Extraction Based on the Normal Vector and Curvature

Double threshold feature extraction based on the normal vector and curvature extracts feature information by using the included angle of the normal vector and the threshold of the average curvature feature weight of points in the local neighbourhood of the point cloud. This method exhibits evident improvement in stability and accuracy due to the constraints of the normal vector and curvature.

The angle of the normal vector is used to construct the constraint condition of feature judgement: the angle between points $p_{i}$ and $p_{j}$ in their *k*-neighbourhood is expressed by $\theta_{ij}$, where $\vec{n_{i}}$ and $\vec{n_{j}}$ are the normal vectors of the $p_{i}$ and $p_{j}$.

(7) $$\theta_{ij}=arccos\frac{\vec{n_{i}}\;\cdot \;\vec{n_{j}}}{\left|\vec{n_{i}}\right|\cdot \left|\vec{n_{j}}\right|},\;\theta_{ij}\epsilon \left[0,\pi \right]$$

The change degree $f_{i}$ of the included angle of the normal vector of point $p_{i}$ is defined as follows:

(8) $$f_{i}=\frac{1}{k}\sum_{j=1}^{k}\theta_{ij}$$

The average curvature local characteristic weight $w_{H}$ of point $p_{i}$ in its *k*-neighbourhood is defined as follows:

(9) $$\{\begin{array}{c} w_{H}\left(p_{i}\right)=\sqrt{\frac{1}{k}\sum_{i=1}^{k}\left(\left|H_{pi}\right|-\bar{H}\right)+\sqrt{\left(H_{i}-\bar{H}\right)}}\\ \bar{H}=\frac{1}{k}\sum_{i=1}^{k}H_{i} \end{array}$$

where H represents the average curvature of point $p_{i}$ in its *k*-neighbourhood. Whether the point belongs to the feature point by $f_{i}$ of the normal vector and $w_{H}$ of the curvature is determined. If $f_{i}$ is greater than the set threshold $\alpha_{1}$, then the point is divided into the potential feature point sets. If $f_{i}$ is less than the set threshold, then it is not a feature point. If $w_{H}$ is greater than the set threshold $\alpha_{2}$, then the point is a feature point, and it is added to the feature point set. If $w_{H}$ is less than the set threshold, then the point is not a feature point, and it is removed from the potential feature point set. Then, the FPFH is used to describe the feature point set to obtain the initial sets of corresponding point pairs A extracted from P and B extracted from Q.

### 2.4. DDM Algorithm

**Figure 4:**
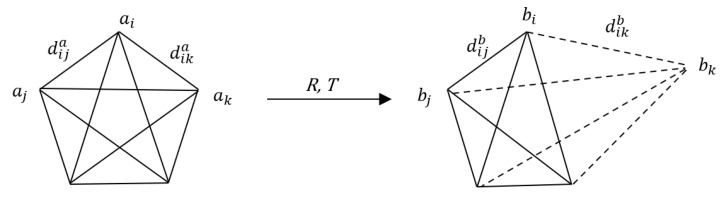
Schematic of wrong point pair.

In rigid body transformation, the distance and principal axis angle of any two feature points remain unchanged before and after transformation. The consistent characteristics of this distance and angle can be used to eliminate unreliable or wrong matching point pairs. Consider the feature point sets$\;A\left\{a_{1},a_{2},\ldots ,a_{n}\right\}$ and $B\left\{b_{1},b_{2},\ldots ,b_{n}\right\}$, where ($a_{i},b_{i}$) represents the corresponding point pair. As shown in Figure 4, the distance between feature points remains basically unchanged before and after transformation; that is, $\left|d_{ik}^{a}-d_{ik}^{b}\right|<e$, and e represents the error threshold. If a mismatch$\;(a_{k},\;b_{k}$) occurs, then the distance between other points in $b_{k}$ and B will change considerably compared with the distance between other corresponding points in $a_{k}$ and A, i.e., $\left|d_{ik}^{a}-d_{ik}^{b}\right|>e$. This difference in Euclidean distance can be used to eliminate external point pairs [33].

The calculation process is as follows:

(1) The distance matrix for point sets A and B is calculated, i.e., $M_{A}=\left[a_{ij}\right]$, $M_{B}=\left[b_{ij}\right]$,(i=1,2,…,n,j=1,2,…n), where $a_{ij}=d\left(a_{i},a_{j}\right)$, $b_{ij}=d\left(b_{i},b_{j}\right)$.
(2) The DDM is calculated, i.e., $M_{DDM}=\left[c_{ij}\right]$, $c_{ij}=\left|a_{ij}-b_{ij}\right|$. Each element in the matrix represents the difference between the Euclidean distances of two feature points before and after rigid body transformation; hence, it is called DDM. If the point pair set has no external value, then $a_{ij}=b_{ij}$, $c_{ij}=0$; otherwise, if ($a_{k},\;b_{k}$) is a wrong match, then a large nonzero value will appear in row k and column k of the symmetric matrix $M_{DDM}$, resulting in ($a_{k},\;b_{k}$) being distinguished from other point pairs.
(3) A vector m is defined to store the mean value of each row of $M_{DDM}$, and the extreme values $m_{max}\;\mathrm{and}\;m_{min}$ are determined. If the subtraction of the two values is less than the set threshold $\alpha_{3}$, then $m_{max}=0$, and the extreme value is continuously found; otherwise, the cycle is exited.

From the preceding calculation process, an out-of-area matching is manifested in $M_{DDM}$ by more nonzero values in its corresponding row, resulting in a large mean $m_{i}$, which is positively correlated with its offset degree. Moreover, $m_{i}$ can reflect the matching accuracy of point pairs ($a_{i},\;b_{i})$. Therefore, wrong matching point pairs can be detected and eliminated by observing vector m. Then the correct sets of corresponding points $A_{C}$ and $B_{C}$ are obtained.

### 2.5. SAC-IA Coarse Registration

To achieve the good registration effect of 3D point clouds, the coarse-to-fine registration strategy is adopted. SAC-IA is used to realise the coarse registration. The principle of the SAC-IA registration algorithm is as follows:

(1) A number of sampling points are selected from the source point cloud P, and the distance between each point should be greater than the minimum distance given in advance to ensure that there are different FPFH between points.
(2) According to the FPFH, one or more points similar to the sampling points are found in the target point cloud Q, and these similar points are regarded as the corresponding points of the sampling points.
(3) The transformation matrix is calculated in accordance with the corresponding points. The performance of registration is evaluated according to the total distance error function by solving the corresponding point transformation, which is expressed as follows:

(10) $$H\left(l_{i}\right)=\{\begin{array}{c} \;\frac{1}{2}l_{i}^{2},\;\;\;\;\;\;\;\;\;\;\;\;\;\;\;\;\;\;\;\;\;\;\;\;|\|l_{i}\|<m_{i}\\ \frac{1}{2}m_{i}\left(2\|l_{i}\|-m_{i}\right),\;\;|\|l_{i}\|>m_{i} \end{array}$$

in which, $m_{i}$ is the specified value and $l_{i}$ is the distance difference after the corresponding point transformation. When the registration process is completed, the one with the smallest error in all the transformations is considered the optimal transformation matrix for initial registration.

In coarse registration, we use the correct sets of corresponding points $A_{C}$ and $B_{C}$ to replace the P and Q, and obtain the initial translation matrix. Perform the initial transformation matrix on the point cloud $A_{C}$, and get the transformed corresponding point cloud $A_{C}^{\prime }$.

### 2.6. ICP Fine Registration

After coarse registration with the SAC-IA algorithm, the source and target point clouds roughly coincide, but the registration accuracy is low. Then, the ICP algorithm is used for fine registration. The specific algorithm steps are as follows:

(1) The point cloud after coarse registration $P^{\prime }$ and the target point cloud Q are taken as the initial point set for fine registration.
(2) For all points $p_{i}^{\prime }$ of $P^{\prime }$, the nearest corresponding point $q_{i}$ in Q is found to form the initial corresponding point pairs.
(3) Use the least square method to solve the optimal rotation matrix R and translation matrix T, perform R, T on the point cloud $P^{\prime }$ and the mean square error (MSE) function $d_{k}$ is minimized.

(11) $$d_{k}=\frac{1}{N}\sum_{i=1}^{N}\|q_{i}-{\left(Rp_{i}^{\prime }+T\right)\|}^{2}$$

(4) Set the threshold ε, if the condition $d_{k}-d_{k+1}<\varepsilon$ is met, end the iteration and get the final transformation matrix R and T.

In fine registration, we use the point cloud $A_{C}^{\prime }$ to replace the $P^{\prime }$. According to the finally obtained R and T, the source point cloud P is transformed into the coordinate system of the target point cloud Q to complete the registration.

## 3. Results

The software environment used in this experiment is Microsoft Visual Studio 2017 and Point Cloud Library running on an AMD Ryzen 7 4800u computer with 16.0 GB and a 64-bit Windows 10 operating system.

### 3.1. Selection of Mian Parameters

To verify the effectiveness of the algorithm, validation experiments are performed using Bunny, Dragon, Armadillo, and Buddha models from Stanford University, and the Airplane model from the Modelnet40 Dataset [34]. To verify the soundness and efficiency of the algorithm proposed in this study, it is compared with the ICP algorithm and the algorithms in [27,28]. MSE is a commonly used error measurement method in point cloud registration; it is the average of the sum of squares distances between the corresponding points of two-point clouds. A smaller MSE indicates a better registration effect. The results of each algorithm are compared in terms of MSE and registration time.

Registration results of the proposed algorithm are related to four main parameters, including the neighbourhood radius *k*, thresholds $\alpha_{1}$, $\alpha_{2}$, and $\alpha_{3}$. Table 1 shows the average number of neighbourhood points under different values of *k*. For each point $p_{i}$, calculate the normal vector and curvature under different values of *k*.

When k=0.001 m, the average number of neighbourhood points is too few and no result is obtained, whilst when k ≥ 0.003 m, too many points lead to long calculation time. Thus, the value of *k* is selected as 0.002 m.

**Figure 5:**
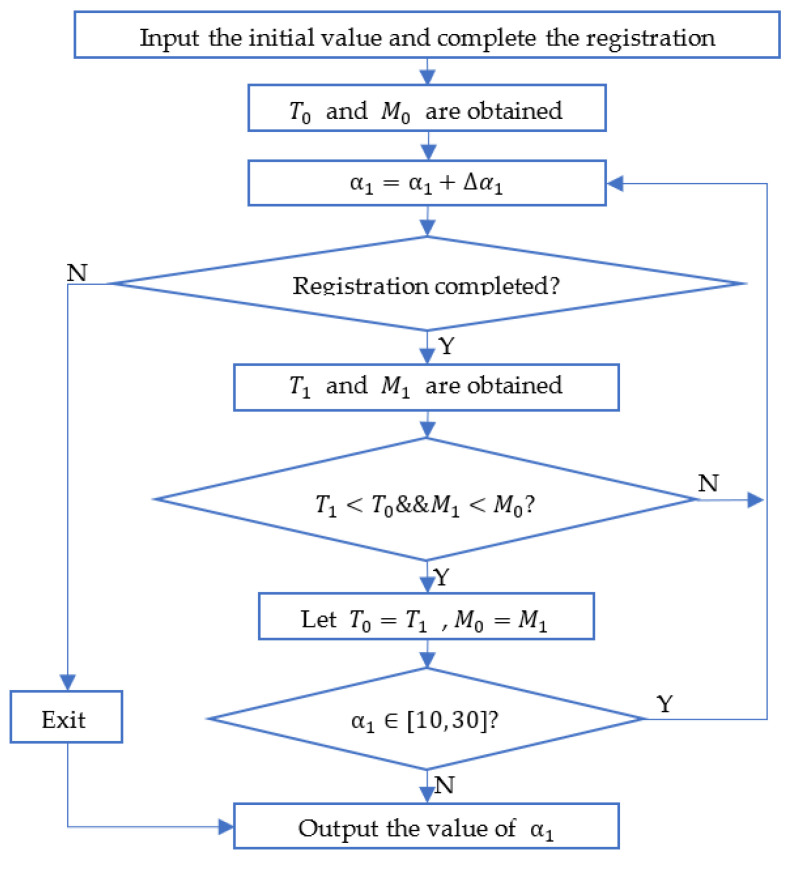
Flowchart of how to obtain the threshold value $\alpha_{1}$.

Based on the calculated results of $f_{i}$, $w_{H}$, and $M_{DDM}$, we set the initial scope of $\alpha_{1}\in \left[10,\;30\right]\;\mathrm{rad}$, $\alpha_{2}\in \left[5,\;20\right]\;1/m$ and $\alpha_{3}\in \left[0.0005,\;0.002\right]\;m$. Figure 5 is the flowchart of how to obtain the threshold value $\alpha_{1}$. First, we input the initial value that $\alpha_{1}=10\;\mathrm{rad}$, $\alpha_{2}=5\;1/m$, and $\alpha_{3}=0.002\;m$ in the proposed algorithm and get the registration results MSE $M_{0}$ and time $T_{0}$. Then, the step size $∆\alpha_{1}=1$ is set. When the threshold value is too large and the number of extracted feature points is too small, the registration fails. The next registration is performed under $\alpha_{1}=\alpha_{1}+∆\alpha_{1}$, and the MSE $M_{1}$ and time $T_{1}$ are obtained. By comparing these two registration results, the $\alpha_{1}$ under the smaller registration result is selected for the next comparison. Finally, we get the value of $\alpha_{1}$ with the smallest registration result in the range. The initial value of $\alpha_{1}$ is modified, and then calculate it in the same way to get the value of $\alpha_{2}$. The step size $∆\alpha_{2}=1\;$ and $∆\alpha_{3}=0.0001$ are set.

The value of all parameters in the proposed algorithm are listed in Table 2.

### 3.2. The Results of Experiments

The experiment is divided into two cases. Case 1: the point cloud under one angle is converted into another angle by the matrix for registration. Case 2: two-point clouds are collected under different angles for registration.

For Case 1, the target point cloud is converted from the source point cloud with the same number of points, and the two-point clouds exhibit one-to-one correspondence without missing points. Case 1 simulates the unique correspondence of feature points under ideal conditions as a way to verify that the feature points extracted by our algorithm are valid and correct. Theoretically, if the feature points are accurate, the two-point clouds can overlap exactly.

**Figure 6:**
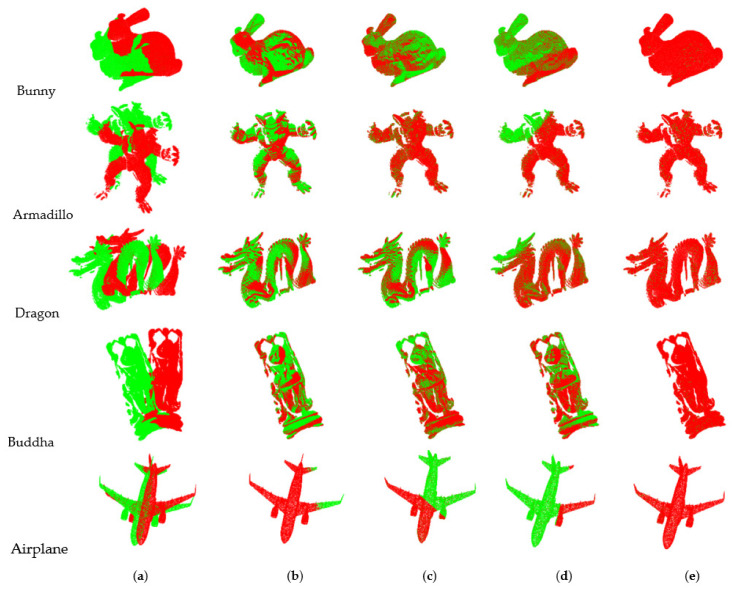
Registration effect of the same angle. (**a**) Initial position; (**b**) ICP; (**c**) Algorithm in [27]; (**d**) Algorithm in [28]; (**e**) Proposed.

Table 3 shows the registration results of each algorithm for the five models in Case 1. ICP takes a long time to process the big data point cloud, has low efficiency, and exhibits poor registration effects. As the number of point clouds increases, the time becomes longer. The algorithms in [27,28] down-sample the initial point cloud and extract feature points by a single feature. The number of the extracted feature points is large and it takes time to complete the coarse registration. Although the registration is completed, the MSE of the two algorithms is large, which indicates that the feature points contain the wrong points. It can also be seen from Figure 6 that there is a position error between the two-point clouds. The MSE of our algorithm is almost zero and the two-point clouds completely overlap, which represents exact registration with accurate feature points.

In the Airplane model, the algorithms in [27,28] spend a lot of time on coarse registration. The reason is that the down-sampling is affected by point cloud density. The proposed algorithm calculates the feature information based on the neighbourhood points without the influence of the point cloud density. For models with particularly complex surfaces, such as the Buddha, many feature points take time in coarse registration, but accurate registration can also be accomplished. In the figures, the target point clouds are in green, the source point clouds are in red.

For Case 2, i.e., the conventional case, there are occlusion, deletion, noise interference, and other factors, which will lead to different point cloud data collected from different views in multi-view 3D scanning. Therefore, the accuracy of feature points plays an important part in registration.

**Figure 7:**
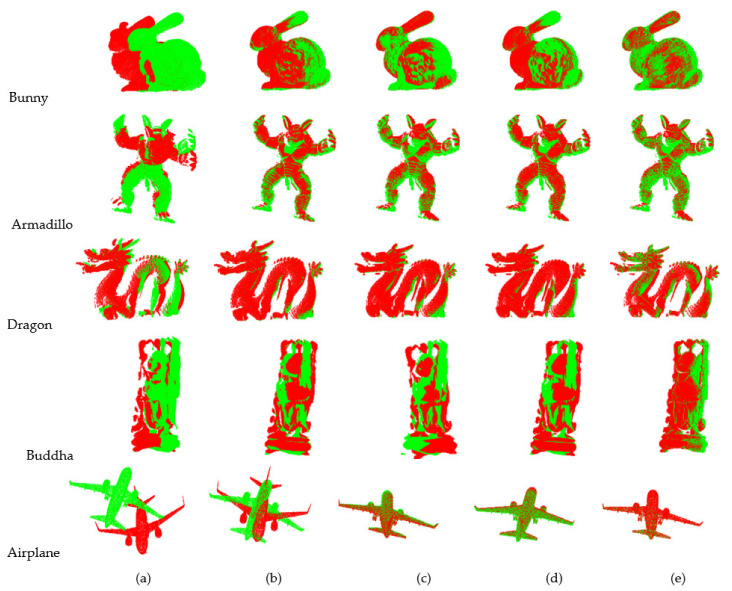
Registration effect of different angles.(**a**)Initial position; (**b**)ICP; (**c**) Ref. [27]; (**d**) Ref. [28]; (**e**) Proposed.

Table 4 shows the registration results of Case 2 in different models. The accuracy of the ICP algorithm and the algorithms in [27,28] is comparable, whilst the proposed algorithm is more accurate. For general surfaces, the registration time of our algorithm is considerably shorter than that of the ICP algorithm, 1/4 of that of the algorithm in [27] and 1/2 of that of the algorithm in [28]. When the point cloud density changes, there are too many points after the down-sample, resulting in a long registration time of the algorithms in [27,28]. The time of our algorithm is not affected by the point cloud density and is still able to complete the registration in a relatively short time. For complex surfaces, although our algorithm takes longer time in registration, the accuracy is high. As can be seen from Figure 7, our registration effect is the best compared to the other three algorithms, especially when registering objects with complex surfaces.

The comparison of the number of point clouds in each processing stage is provided in Table 5. After the double threshold feature extraction of the original point cloud, the number of point clouds is considerably reduced. After the feature description and finding the corresponding point pairs, the mismatched point pairs are removed by the DDM algorithm, and the number of finally registered point clouds is about 0.1% of the original point clouds. Therefore, the selection of highly representative feature points is a prerequisite for high-precision registration. Compared with those of the traditional ICP and improved algorithms, the efficiency and accuracy of our proposed algorithm are significantly improved.

The algorithm proposed in this study uses neighbourhood point information to extract feature points, eliminate mismatched point pairs, reduce the number of point cloud registrations, and improve the correspondence between point pairs, which is suitable for objects that have distinctive features on the surface. The accuracy of the registration is not affected by the density of the point cloud, but the registration time is related to the complexity of the surface. Compared with those obtained via down-sampling, feature points extracted on the basis of the normal vector and curvature are more representative and can achieve high-precision point cloud registration.

## 4. Conclusions

In the process of point cloud registration, feature points affect the registration results, which represent the big data point cloud, and can improve the efficiency of the algorithm. In addition, the descriptor can determine the final performance. It can provide a useful representation of the shape around the fixed point and help search for the corresponding relationship between the two shapes, avoiding an exhaustive search. The combination of the two can considerably improve the efficiency and accuracy of registration.

In the current study, we proposed an improved registration algorithm based on double threshold feature extraction and DDM. We studied the translation-rotation invariance of point cloud geometric features during rigid body transformation, and we extract feature points with double threshold by using normal vector and curvature. Wrong points may occur in feature extraction under a single condition. The double constraints of the normal vector and curvature make extracting more representative feature points possible. It also greatly reduces the number of original point clouds and reduces the number of subsequent calculations. Some feature points extracted via down-sampling cannot represent the feature of this region, and the probability of wrong point pairs is relatively high. FPFH is applied to describe feature points and find initial matching point pairs quickly. According to the principle of the DDM algorithm, it can effectively remove the mismatched point pairs of the initial matching and further reduce the number of point clouds. The final number of the point cloud is just 0.1% of the original point cloud. Through the above processing, we not only improve the accuracy of the feature points, but also gradually reduce the amount of data to be processed at the same time, which paves the way for subsequent registration.

However, the final corresponding point pairs cannot be guaranteed to be completely correct and influence the final registration results due to noise and other interference factors. Besides, the registration time of this algorithm is related to the complexity of the object surface. Too many feature points lead to a longer registration time. In the next step, we will first perform noise processing on the original point cloud data and investigate feature descriptors to improve the accuracy of the corresponding point pairs to help make registration more precise.

## Figures and Tables

**Table 1:** The average number of neighbourhood points under different values of *k*.

k/m	0.01	0.0009	0.0008	0.0007	0.0006	0.005	0.004	0.003	0.002	0.001
Neighbourhood points	623	507	402	309	229	159	102	58	25	5

**Table 2:** All parameters of the proposed algorithm.

Parameters	Value	Definition
k	0.002 m	Neighbourhood radius
$\alpha_{1}$	20 rad	Threshold of normal vector change degree
$\alpha_{2}$	15 1/m	Threshold of average curvature local characteristic weight
$\alpha_{3}$	0.001 m	Threshold of ending the DDM algorithm
$R_{FPFH}$	0.004 m	Radius of calculating FPFH
$\varepsilon_{icp}$	0.01 m	Max distance threshold of corresponding points for ICP
$Iter_{icp}$	50	Max iteration number for ICP

**Table 3:** Registration results from the same angle.

Model	Number of Points	Algorithm	Time of Coarse Registration	Time of ICP	MSE
Bunny (0°)	40,256	ICP	/	61.758 s	1.939 × 10^−7^
		Algorithm in [27]	11.805 s	1.088 s	2.560 × 10^−7^
		Algorithm in [28]	3.443 s	0.652 s	9.116 × 10^−7^
		Proposed	1.676 s	0.604 s	1.259 × 10^−16^
Armadillo (60°)	23,404	ICP	/	20.287 s	5.169 × 10^−7^
		Algorithm in [27]	8.593 s	0.663 s	3.041 × 10^−7^
		Algorithm in [28]	2.839 s	0.412 s	8.219 × 10^−8^
		Proposed	2.807 s	0.345 s	3.515 × 10^−16^
Dragon (24°)	34,836	ICP	/	56.146 s	2.735 × 10^−7^
		Algorithm in [27]	10.561 s	0.908 s	3.967 × 10^−7^
		Algorithm in [28]	4.833 s	0.616 s	9.306 × 10^−8^
		Proposed	1.610 s	0.511 s	8.385 × 10^−16^
Buddha (0°)	78,056	ICP	/	164.677 s	5.632 × 10^−8^
		Algorithm in [27]	30.416 s	4.158 s	8.658 × 10^−8^
		Algorithm in [28]	4.098 s	1.418 s	4.228 × 10^−8^
		Proposed	20.666 s	2.873 s	6.643 × 10^−16^
Plane	36,980	ICP	/	39.74 s	5.185 × 10^−12^
		Algorithm in [27]	324.319 s	3.639 s	5.932 × 10^−7^
		Algorithm in [28]	132.853 s	1.103 s	1.904 × 10^−7^
		Proposed	1.146 s	0.539 s	1.835 × 10^−15^

**Table 4:** Registration results of different angles.

Model	Number of Points	Algorithm	Time of Coarse Registration	Time of ICP	MSE
Bunny (0° and 45°)	40,256, 40,097	ICP	/	47.315 s	9.332 × 10^−6^
		Algorithm in [27]	6.141 s	1.261 s	1.302 × 10^−6^
		Algorithm in [28]	2.900 s	0.737 s	2.995 × 10^−5^
		Proposed	0.991 s	0.599 s	4.481 × 10^−8^
Armadillo (60° and 90°)	23,404, 28,341	ICP	/	19.867 s	6.527 × 10^−6^
		Algorithm in [27]	11.957 s	0.815 s	2.230 × 10^−6^
		Algorithm in [28]	3.580 s	0.519 s	4.474 × 10^−6^
		Proposed	1.558 s	0.427 s	6.581 × 10^−8^
Dragon (24° and 48°)	34,836, 22,092	ICP	/	27.818 s	1.559 × 10^−5^
		Algorithm in [27]	3.385 s	0.709 s	6.722 × 10^−6^
		Algorithm in [28]	4.701 s	0.531 s	2.325 × 10^−5^
		Proposed	1.789 s	0.348 s	9.180 × 10^−8^
Buddha (0° and 48°)	78,056, 69,158	ICP	/	224.568 s	2.223 × 10^−5^
		Algorithm in [27]	9.837 s	2.454 s	2.159 × 10^−5^
		Algorithm in [28]	3.789 s	1.511 s	2.158 × 10^−5^
		Proposed	10.260 s	1.137 s	7.440 × 10^−8^
Airpane	36,980, 34,117	ICP	/	165.056 s	2.235 × 10^−4^
		Algorithm in [27]	274.603 s	6.119 s	6.248 × 10^−6^
		Algorithm in [28]	49.362 s	2.317 s	5.254 × 10^−6^
		Proposed	0.910 s	0.491 s	2.037 × 10^−8^

**Table 5:** Comparison of numbers in each processing stage.

Point Clouds	Number of Points			
	Original Points	Feature Points	Initial Matched Points	Final Matched Points
Bunny 0° and 45°	40,256, 40,097	1044, 487	31	15
Armadillo 60° and 90°	23,404, 28,341	2324, 2711	73	38
Dragon 24° and 48°	34,836, 22,092	1441, 1228	79	49
Buddha 0° and 48°	78,056, 69,158	7436, 5648	884	375
Airplane	36,980, 34,117	1162, 879	15	10

## Data Availability

Not applicable.
